# Age-Dependent Differences in Systemic and Cell-Autonomous Immunity to *L. monocytogenes*

**DOI:** 10.1155/2013/917198

**Published:** 2013-04-07

**Authors:** Ashley M. Sherrid, Tobias R. Kollmann

**Affiliations:** Division of Infectious & Immunological Diseases, Department of Pediatrics, University of British Columbia, CFRI Rm. A5-147, 938 West 28th Avenue, Vancouver, BC, Canada V5Z 4H4

## Abstract

Host defense against infection can broadly be categorized into systemic immunity and cell-autonomous immunity. Systemic immunity is crucial for all multicellular organisms, increasing in importance with increasing cellular complexity of the host. The systemic immune response to *Listeria monocytogenes* has been studied extensively in murine models; however, the clinical applicability of these findings to the human newborn remains incompletely understood. Furthermore, the ability to control infection at the level of an individual cell, known as “cell-autonomous immunity,” appears most relevant following infection with *L. monocytogenes*; as the main target, the monocyte is centrally important to innate as well as adaptive systemic immunity to listeriosis. We thus suggest that the overall increased risk to suffer and die from *L. monocytogenes* infection in the newborn period is a direct consequence of age-dependent differences in cell-autonomous immunity of the monocyte to *L. monocytogenes*. We here review what is known about age-dependent differences in systemic innate and adaptive as well as cell-autonomous immunity to infection with *Listeria monocytogenes*.

## 1. Introduction

*L. monocytogenes* is an opportunistic pathogen that mainly affects very young, old, or immune compromised individuals [1]. Epidemics of listeriosis are associated with high mortality rates and continue to cause widespread concern [2–9]. The fact that the newborn in particular suffers a much higher risk of severe outcome suggests that deficiencies exist in the host defense of the newborn versus the young adult against *L. monocytogenes* [10].

Host defense against infection can broadly be categorized into systemic immunity and cell-autonomous immunity [11, 12]. Systemic immunity is crucial for all multicellular organisms, increasing in importance with increasing cellular complexity of the host. Differences in innate as well as adaptive systemic immunity between the neonatal versus adult host in response to *L. monocytogenes* infection undoubtedly contribute to their difference in clinical response, and they are summarized here [10, 13–17]. However, from plants to humans, the ability to control infection at the level of an individual cell equates firmly with survival of the host [18]. This capacity for cell intrinsic self-defence is called cell-autonomous immunity [12]. Cell-autonomous immunity is operationally defined by a minimal set of genetically encoded antimicrobial defense factors that enables an infected host cell to resist a pathogen [18]. In higher organisms, cell-autonomous immunity following microbial exposure is characterized by the rapid induction of a transcriptional program [19]. Successful execution of this defense program is necessary for the survival of not only the single cell but also the host [18]. We postulate that much of the increased risk to suffer and die from *L. monocytogenes* infection in the newborn period is a consequence of age-dependent differences in cell-autonomous immunity to *L. monocytogenes*.

## 2. Systemic Immunity to Listeriosis

The increased susceptibility of neonates to suffer from severe listeriosis is a well-documented clinical phenomenon. However, the mechanisms leading to this susceptibility are only incompletely understood. *Listeria monocytogenes *has been used extensively in mouse infection models to elucidate the inner workings of the immune system in response to pathogenic challenge. While mice mimic certain aspects of human immunity and pathogen susceptibility, the model has certain limitations, and it is unknown how closely it parallels clinical susceptibility to *L. monocytogenes. *Our knowledge about the human response to *Listeria *infection is confined primarily to results obtained from *in vitro *experiments. Elucidation of the ontogeny of host innate and adaptive immune development [20, 21] has also added to our conceptual understanding of age-dependent differences in immunity; however, their relevance to infection with *Listeria *is not clear. In this section, we will detail the key contributing effectors of the host systemic innate and adaptive immune response to *Listeria, *weaving together information from mechanistic studies in animal infection models and human studies in primary cells.

### 2.1. Innate Immune Response

#### 2.1.1. Innate Immune Response in the Mouse

The first line of defense against *Listeria *is the gastrointestinal barrier. Within intestinal crypts, Paneth cells produce antimicrobial effectors including lysozyme, phospholipase A2, and alpha defensins. *L. monocytogenes *infects intestinal epithelial cells and is also taken up from the intestine through Peyer's patches and macrophages of the lamina propria. From there, bacteria disseminate to the liver, spleen, and mesenteric lymph node through the blood and lymph [22], often carried within host monocytes [23].

Within these tissues, bacteria are initially taken up by resident macrophages, which produce chemokines to promote recruitment of monocytes and neutrophils to the site of infection. Recruitment of monocytes to sites of infection is central to the early control of murine *L. monocytogenes *infection, as shown by the increased susceptibility of mice lacking CCR2 or CCL2, the receptor and ligand for monocyte recruitment [24, 25]. Following migration, monocytes differentiate locally into macrophages and a subset of TNF/iNOS producing dendritic cells (TipDCs) [26]. Infected macrophages secrete TNF-*α*, IL-12p70, and IL-18, cytokines that activate NK cells and CD8+ “bystander” T cells to produce IFN-*γ* [27–30]. IFN-*γ* production at early time points is required to activate macrophages in order to kill intracellular bacteria. NK cells have typically been regarded as the primary early producers of IFN-*γ* in the mouse, but this assumption has been called into question by evidence that “bystander” CD8+ T cells can produce IFN-*γ* at early time points in an antigen-independent manner. In fact, based on transfer of NK or CD8+ T cells into IFN-*γ*-deficient recipient mice, CD8+ T cells provide more effective “bystander” protection than NK cells [30, 31]. IFN-*γ* is also required for the differentiation of murine monocytes into TipDCs, though NK cells appear to be the primary source of IFN-*γ* for this differentiation process [32]. During *L. monocytogenes *infection of mice, CD11b^+^ CD11c^int^ myeloid lineage cells are the main source of TNF-*α* and iNOS, which are both crucial mediators of the murine anti-*Listeria *response [26, 33, 34]. Cells of the myeloid lineage, such as TipDCs, are also primary producers of IFN-*β* following *L. monocytogenes *infection in mice [26, 35, 36]. As will be discussed in a later section, high levels of type 1 IFNs (IFN-*α* and IFN-*β*) have been implicated in promoting apoptosis of several cell types, and mice deficient for the type 1 IFN receptor are more resistant to *L. monocytogenes *[37, 38].

In mice, the immediate wave of neutrophil migration, which occurs between 30 minutes and 4 hours after infection, is driven by the production of formyl peptides [39]. Following migration into the tissues, neutrophils kill extracellular bacteria through secretion of bactericidal granules and neutrophil extracellular traps (NETs); this appears to be of greater importance in the mouse liver than the spleen [40]. However, the role of neutrophils in defense against *L. monocytogenes* remains somewhat controversial as initial neutrophil depletion studies suggested essentiality of these cells in early infection, but the antibody used has since been found to bind inflammatory monocytes as well as neutrophils [40]. More recent studies utilizing the murine neutrophil-specific Ly6G-specific 1A8 antibody indicate that depletion of neutrophils prior to infection causes 10–1000-fold higher *Listeria *burdens within the first 3 days of infection, while initiation of neutrophil depletion alongside infection has no effect [41, 42]. These data suggest that neutrophils primarily contribute to controlling *L. monocytogenes *early during infection.

Dendritic cells (DCs) are key for antigen presentation to T cells, priming of T cells, and cytokine production in the response to *L. monocytogenes*. In mice, conventional DCs (cDCs) undergo maturation following phagocytosis of *L. monocytogenes*. Within the cDC subset, CD8*α*+ DCs contain the highest bacterial burden, generate high levels of IL-12, and are particularly potent at priming T cell responses [43–45]. CD8*α*+ DCs are proficient at cross-presentation of antigens from phagocytosed material including dead or dying cells, via the MHC-I pathway [46], while CD8*α*− DCs are central to presentation through MHC-II class molecules [47]. Additionally, CD8*α*+ DCs have also been implicated in providing intracellular transport of bacteria from the marginal zone to the periarteriolar lymphoid sheath (PALS), where *L. monocytogenes *grows profusely and causes lymphocyte apoptosis [48]. This was further demonstrated by marked resistance to *Listeria *in mice deficient for the transcription factor Batf3, which specifically lack CD8*α*+ DCs. Thus, DCs are crucial in activating *Listeria-*specific T cells but possibly also contribute to early containment of bacterial replication.

#### 2.1.2. Innate Immune Response in the Human

Very little is known about the human systemic innate immune response in listeriosis. Following ingestion of *L. monocytogenes *in contaminated food, bacteria are known to mediate uptake into human epithelial cells through interaction of the protein internalin A with the host protein E-cadherin [49, 50]. This mechanism of oral infection is not conserved in mice due to a single polymorphism in E-cadherin, which renders mice highly resistant to oral listeriosis [51]. Experiments in other models including the guinea pig have begun to reveal fundamentals of bacterial uptake and dissemination following oral *L. monocytogenes *infection, but the availability of tools for these models remains limited [22]. Much remains to be done in order to elucidate *L. monocytogenes *pathogenesis immediately after oral ingestion, utilizing models that utilize either humanized mice or murinized *L. monocytogenes *to allow dissection of mechanisms relevant for bacterial uptake from the gastrointestinal tract [52–54]. *In vitro *models of infected human primary cells and cell lines have indicated the likely response of some key cell types to *L. monocytogenes; *however, these experiments give no indication of the relative importance or specific role played by host cells *in vivo* in human listeriosis. Clinical susceptibility of individuals with genetic-, infection-, or medication-induced immunodeficiencies has provided some insights. For example, an increased risk for severe listeriosis is noted among individuals receiving immunosuppressive medications that interfere with cell-mediated immunity and production of TNF-*α* [28, 55, 56].

#### 2.1.3. Innate Immune Response in the Neonatal Mouse Model

Our knowledge about neonatal listeriosis is severely limited, despite the fact that this age group suffers so severely from this infection. A much lower dose of *L. monocytogenes* is required to result in systemic infection in newborn rather than in adult mice; however within the first two weeks of life, newborn mice gradually develop adult-level resistance to *L. monocytogenes *[57]. Heightened susceptibility of neonatal mice is also noted if they are infected systemically [58]; therefore, age-dependent differences within the gastrointestinal tract are unlikely to be the sole cause for the increase in neonatal susceptibility to severe listeriosis. In mice, neonatal susceptibility correlates with delayed systemic production of innate cytokines and activation of NK cells [57, 58]. At birth, mice have dramatically fewer CD8*α*+ DCs and much lower IL-12 production in response to antigen. These levels gradually increase, reaching adult levels sometime after day 10 of life [59]. In a murine neonatal listeriosis model, splenocytes from infected neonates showed reduced transcription of T-helper-type-I (Th1-) supporting cytokines (IL-12p70 and IFN-*γ*) following restimulation, as compared to infected adults [60]. Neonatal mice also produced elevated levels of the cytokine IL-10 compared to adults upon infection with *L. monocytogenes *[61], and the survival-increasing and CFU-reducing benefits of IL-10 blockade were of substantially longer duration and of enhanced effect in neonates. Interestingly, it was shown that activation of phagocytes with IFN-*γ* prior to infection substantially increased resistance of newborn mice to *L. monocytogenes* [58, 62]. Monocyte chemotaxis to the site of infection is also delayed in neonatal mice [63]. These findings cumulatively suggest that neonates generate an altered innate cytokine response to *L. monocytogenes *infection in comparison with adults. While these differences likely contribute to neonate susceptibility, the mechanisms responsible and their applicability to human infection are not yet clear.

#### 2.1.4. Innate Immune Response in Human Neonates

For the human neonate we can only extrapolate from general concepts of innate immune ontogeny to possible mechanisms leading to age-dependent differences in susceptibility to *Listeria* infection. For example, adhesion and chemotaxis (directed migration) of human neonatal neutrophils and monocytes are markedly deficient in comparison to adult cells [64, 65]. Furthermore, innate cytokine responses of neonates markedly differ from those of adults. For example, TLR-induced generation of proinflammatory cytokines such as TNF-*α* and IL-1*β* differ in the neonate depending on the stimulant, reaching adult-level production between 1-2 years of age. During this time period, production of IL-10, IL-6, and IL-23 undergoes a slow decline from a perinatally higher than adult level [20, 21]. And while significantly reduced at birth, the ability of TLR agonists to induce type I IFN production reaches adult-like levels within only a few weeks of life. The last group of TLR-induced cytokines to reach adult-level production is the Th1-supporting innate cytokines IFN-*γ* and IL-12p70 [20, 21, 66–69]. 

These patterns are noteworthy because IFN-*γ*, IL-12p70, and TNF-*α* have key protective roles in the murine innate immune defense against *Listeria, *while IL-10, which neonates make more of, has been shown to increase susceptibility to *Listeria *infection in mice [70, 71]. The low production of type I IFNs in neonates versus adults is notable as well; however, the age-dependent difference here is opposite of what might have been expected based upon the available data. In animal models, type I IFN appears to be detrimental, and *in vitro *studies of human primary cells indicate that high levels of type I IFN promote cell death in several cell types central to *Listeria *defense, as will be discussed in a later section. Thus, the precise impact of low type I IFN production in human neonates is not yet known.

### 2.2. Adaptive Immune Response

Effectors of the innate immune system are capable of controlling infection only over the short term in mice; in fact, SCID mice (deficient for B and T cells) are capable of restraining infection [72] but cannot achieve sterilizing immunity. Thus, the innate immune system must also activate the adaptive immune system for final and complete clearance of *Listeria*. The murine adaptive immune response peaks about 1 week after infection with *L. monocytogenes. *It has been demonstrated in mouse infection models that T cell responses are central to clearance of *L. monocytogenes *infection, with humoral responses playing only a minimal role [29, 73]. As described above, antigen presentation through both the MHC-I and MHC-II pathways is primarily mediated by DCs, activating CD8+ and CD4+ T cells specific for *Listeria *antigens, respectively [44]. Of the two, CD8+ cytotoxic T cells play a more important role in control of listeriosis than CD4+ cells [74], though the relative importance of several known potential mechanisms of protection is still a matter of debate. The innate cytokine IL-12p70 is important for the expansion phase of the CD8+ T cell response [75]; IL-12p70 appears to activate T cells into full effector cells necessary for control of *L. monocytogenes* infection. The role of CD4+ T cells requires IFN-*γ* production by these cells and likely involves the reciprocal activation of macrophages [76]. CD4+ cells appear to be important for the initial stage of CD8+ T cell priming and for memory longevity [29, 77, 78]. Murine *γδ* T cells are also known to play a role in IFN-*γ* production during infection [79]. While it is not known how closely the mouse model mimics the adaptive immune response to clinical listeriosis in the human, the susceptibility of individuals with AIDS or those undergoing treatment to suppress cell mediated immunity indicates that T cells likely perform a central role in human defense against listeriosis as well [28].

Some crucial mediators of adaptive immune defense against *Listeria *appear to differ qualitatively or quantitatively in neonates. At birth, neonatal CD4+ T cells in mice appear to be Th2 biased [80]. In addition, neonatal CD4+ Th1 cells have been shown to undergo apoptosis when reexposed to antigen, whereas Th2 cells do not [81]. Another potential difficulty of the neonatal response to infection stems from the fact that murine lymphoid cells are limited in number early in life; therefore, a suitable expansion of cells could be difficult to attain [82]. Finally, the reduced production of innate IL-12p70 and increased production of IL-10 by neonatal innate cells upon stimulation would be expected to lead to suboptimal activation of CD8+ T cells and thus increased susceptibility to listeriosis [10, 20, 21, 83, 84]. The human adaptive response to neonatal listeriosis has not been adequately examined.

In summary, differences in innate immunity between neonate and adult have been defined [10]; however, few of these differences correlate well with the high-risk period for human neonatal listeriosis typically restricted to the first 6–8 weeks of life [85]. It thus appears likely that factors other than age-dependent differences in innate immune function must also play a role in the increased susceptibility of the human newborn to severe infection with *L. monocytogenes*. While differences for the human newborn versus adult adaptive immune response have been defined [17], the human is already capable of initiating and sustaining strong, protective Th1-type responses prior to birth [86]. Thus again, age-dependent differences in adaptive immunity alone cannot explain the overall increased risk for severe outcome of infection with *L. monocytogenes* early in life. Containment of infection ultimately depends on the interaction between the intracellular *L. monocytogenes* and the infected host cell. The next section will cover this primary battleground.

## 3. Cell-Autonomous Immunity: The Cell as a Battleground

Cell-autonomous immunity is defined as the ability of a single cell to resist infection, while systemic immunity is expressed as resistance of the entire host to infection, that is at the organismal level. For infection with *L. monocytogenes* the differentiation between systemic immunity and cell-autonomous immunity is not as clear, as one of the main target cells infected by *L. monocytogenes* is the monocyte, which is an integral part of the innate immune system, and also the effector arm of the adaptive immune system. For example, as outlined above, T cell interactions with monocytes are critical for survival of the host following *L. monocytogenes* infection. However, T cells do not kill *Listeria*; rather, T cells only lyse infected cells [14], in the process releasing viable bacteria [87]. The main function of the T cell in defense against *L. monocytogenes* instead is to support the monocyte response. Elegant experiments conducted in mice decades ago already clearly identified that age-dependent susceptibility to primary infection with *L. monocytogenes* correlates best with age-dependent differences in monocyte function [57, 58]. Since then, we have learned that for the host not to succumb to *L. monocytogenes*, phagocytes such as monocytes/macrophages have to rapidly trap and kill the ingested bacteria [57, 87–89]. We now also know that, from the moment *L. monocytogenes *binds the monocyte, a response is set into motion that aims to destroy the bacteria [90]. In adult mice, this cell autonomous immune response of the monocyte has been found to be essential for protection from severe listeriosis [32, 87, 91, 92]. This strongly suggests that age-dependent differences in systemic immunity are the result of age-dependent differences in cell autonomous immunity of human monocytes to *L. monocytogenes*. Given the importance of cell autonomous immunity for neonatal infectious disease, it is remarkable how often this form of somatic self-defence is either overlooked or under-appreciated [18]. This is particularly true for listeriosis. In this section, we review what is known about age-dependent differences in the cell autonomous immune response of the monocyte to *L. monocytogenes*.

### 3.1. Monocyte Recognition of *Listeria*

*L. monocytogenes *is recognized by monocytes via several distinct pathways, each setting in motion a host cellular response that involves hundreds of genes [93–95].

- The extracellular and phagosomal Toll-like receptor (TLR)/MyD88-dependent recognition pathway induces expression of inflammatory cytokines such as tumor necrosis factor-*α* (TNF-*α*) as well as reactive oxygen (ROS) and nitrogen (RNS) species in order to kill ingested *L. monocytogenes* [96–101]. Multiple *L. monocytogenes* ligands that are recognized at both the host cell surface and within a vacuole contribute to the MyD88-dependent response to *L. monocytogenes* [13]. This pathway is clearly important for host resistance as we and others have shown that MyD88-deficient mice are extremely vulnerable to *L. monocytogenes* infection [15, 102]. While TLR/MyD88 sensor function appears well developed early in life [103], downstream effector responses are strikingly different in the human newborn as compared to the young adult [10]. As discussed in the previous section, TLR-induced cytokine generation differs between neonates and adults. Additionally, MyD88-induced production of ROS or RNS is also strikingly reduced inearlyas compared to adult life [104–107]. This suggests that the activity of multiple MyD88-dependent effector mechanisms essential for protection from severe infection with *L. monocytogenes * is functionally altered early in life. The period between birth and 6 weeks of age represents the highest risk period for severe infection with *L. monocytogenes * in the human newborn. This period best correlates with the period of low type I IFN production following TLR/MyD88-dependent stimulation, suggesting a possible functional connection [10]. However, the TLR/MyD88 dependent response of human neonatal monocytes to *L. monocytogenes* has not yet been investigated.
- The cytosolic STING/IRF3-dependent pathway in mice leads to the robust expression of interferon-*β* (IFN-**β**) and other interferon stimulated genes (ISG) controlled by the transcription factor IRF3 [108]. Induction of IFN-**β** by cyclic dinucleotides secreted by cytosolic *L. monocytogenes* is entirely STING dependent *in vitro* and *in vivo* [109, 110], as STING functions as the direct host receptor for cyclic dinucleotides [111]. To our knowledge, the developmental pattern of the cytosolic pathway has not been examined in any detail in human monocytes. In mice, IFN-**β**-mediated signals can be harmful or protective for the *L. monocytogenes*-infected mouse, depending on the relative activity of concomitant TLR/MyD88 signalling [87]. In mice, production of IFN-**β** during *L. monocytogenes * infection appears restricted to monocytes and macrophages, with no induction of expression in lymphocytes, neutrophils, or dendritic cells [35]. Cell-type specific differences in IFN-**β** production in response to *L. monocytogenes* infection have not been examined in humans. It is however important to note that while IRF3-dependent production of type-1 IFN in human newborns is reduced as compared to adults [10], production of IFN-**β** in humans in response to *L. monocytogenes* is not dependent on IRF3 (as it is in the mouse) but appears p38 MAPK-dependent [112, 113]. Thus, the role of this pathway for human neonatal listeriosis is not clear.
- Activation of the inflammasome pathway by *L. monocytogenes* leads to proteolytic release of IL-1**β** and possibly to inflammatory cell death called pyroptosis [114, 115]. In mice, *L. monocytogenes* can activate the inflammasome via three different cytosolic sensors: NLRP3, NLRC4, and/or AIM2 [115–124]. Murine IFN-induced GTP-binding protein 5 (GBP5) binds NLRP3 subunits and assembles them into a functional complex during *L. monocytogenes* infection of IFN-*γ*-activated murine macrophages (reviewed by [18]). However, inflammasome activation in response to *L. monocytogenes* has also been described as NLRP3 independent, partially NLRC4 dependent, and fully AIM2 dependent [115]. Alum, the most common vaccine adjuvant, exerts part of its function via activation of the inflammasome [125]. Alum-induced responses significantly decline over the first 2 years of life [126], suggesting age-dependent differences in at least some inflammasome activities. However, the developmental pattern of the various inflammasome pathways in humans in response to *L. monocytogenes* has not been elucidated. The importance of the inflammasome pathway for age-dependent susceptibility to *L. monocytogenes* thus is not known.

### 3.2. Fate of* Listeria* inside the Monocyte

Entry of *L. monocytogenes *into monocytes/macrophages occurs via phagocytosis [43, 127]. This process is initiated after *Listeria* is bound by complement that together with the listerial protein internalin B functions as ligands for complement receptors on phagocytes. In addition, scavenger receptors recognize lipoteichoic acid, a component of the listerial cell wall [128]. Once bound by either scavenger or complement receptors, the bacteria are internalized into a phagosome. The phagosome then undergoes a series of transformations via sequential interaction with subcompartments of the endocytic pathway, eventually maturing into a phagolysosome. During this process, engulfed bacteria are exposed to a range of pH-dependent host microbicidal effectors that include ROS and RNS, iron scavengers and exporters, lactoferrin and natural resistance-associated macrophage protein 1 (NRAMP1), antimicrobial peptides and proteins (e.g., defensins, cathelicidins, lysozyme as well as other carbohydrate hydrolases, phospholipases, and various proteases and peptidases) that permeabilize and degrade the ingested bacteria. Production of several of these key molecules has been found reduced in early life [129]; however, precise roles have not been ascribed to any with respect to human or murine neonatal infection with *L. monocytogenes*.

The ability to escape from the phagosome enables *L. monocytogenes* to avoid certain destruction and to instead replicate in the cytosol [130]. This phagosomal escape can occur as rapidly as 30 min after bacterial cell entry [130–132]. The escape of *L. monocytogenes* from the single-layer membrane vacuoles is assisted by virulence-associated bacterial molecules (listeriolysin O (LLO) and phosphatidylinositol-phospholipases (e.g., PC-PLC and PI-PLC)), as well as several host derived factors, such as the *γ*-interferon-inducible lysosomal thiol reductase (GILT) [133, 134]. While LLO is absolutely required for phagosome vacuolar escape in mice, it is dispensable in human cells, where the phospholipases are critical instead [135].

The intracellular fate of phagocytosed *L. monocytogenes* depends on the speed of phagosome maturation versus listerial escape. This dynamic host-pathogen interactive process [130] has not been examined at all in human neonates. From studies in the murine host we know that IFN-inducible GTPases are centrally involved in restricting listerial escape from the phagosome [11]. At least two families of IFN-inducible GTPases—the 21–47 kDa immunity-related GTPases (IRGs) and the 65–73 kDa GBPs—regulate intracellular traffic of phagosomes containing bacteria. Over 20 IRGs have been identified in mice, while the human genome only contains two (reviewed by [18]). Murine Irgm1 is known to target the early *L. monocytogenes* phagosome, where it directs trafficking of bacteria-containing phagosomes and endosomes along microtubules towards maturing phagolysosomes. And the IFN-*γ*-induced guanylate-binding protein 7 (Gbp7) is known to direct the assembly and activation of ROS producing NOX2 holoenzymes specifically on phagosomes containing *L. monocytogenes *[11].

At least four other murine Gbps—Gbp1, Gbp6, Gbp7, and Gbp10—confer cell-autonomous immunity to listerial infection [136]. Mice deficient in Gbp1 display significantly increased susceptibility to *L. monocytogenes *[136]; this systemic *in vivo* phenotype is directly attributable to a role for Gbp1 in cell-autonomous immunity of the macrophage, resulting in delayed and reduced transport of antimicrobial peptides, autophagic machinery, and components of the NADPH oxidase to the phagosomal compartments that contain *L. monocytogenes *(reviewed by [18]). Identification of interacting partners for Gbps has begun to reveal some of the specific molecular mechanisms involved in Gbp-mediated listerial killing (reviewed by [11, 18]). Gbp1 interacts with the ubiquitin-binding proteins, delivering ubiquitinated *L. monocytogenes* to autolysosomes. Gbp7 recruits the autophagy protein ATG4B, which drives the extension of autophagic membranes around bacteria within damaged bacterial compartments and assembles NOX2 on these compartments. And as mentioned above, Gbp5 binds NLRP3 to promote specific inflammasome responses during the infection of IFN-*γ*-activated murine macrophages by *L. monocytogenes*. Gbps thus seem essential for cell-autonomous immunity of the murine monocyte/macrophage to *L. monocytogenes *[137]. Unfortunately, nothing at all is known about either expression or function of GBPs in human neonatal monocytes.

Autophagy is a process by whichcytoplasmic materials,including bacteria, are targeted to lysosomes for degradation (reviewed in [19, 138, 139]). Autophagy has been shown to target *L. monocytogenes* within intact phagosomes, damaged phagosomes, and those found in the cytosol [140]. Therefore, *L. monocytogenes* must successfully evade killing by the autophagy system at all stages of its residence within host cells. *L. monocytogenes* has developed strategies to prevent being taken up by the autophagosome. For example, ActA recruits host proteins to disguise *L. monocytogenes* from ubiquitination and thus prevent autophagic recognition [141, 142]. InlK is another surface protein that contributes to listerial escape from autophagy [143] via recruiting the major vault protein (MVP) to evade ubiquitination and autophagic recognition [138, 144]. In murine cells, expression of LLO is necessary for the induction of the autophagic response, specifically at the early time points after infection; this suggests a role for permeabilization of the vacuole in the induction of the autophagic pathway. However, it is the expression of the phospholipases that allows *L. monocytogenes* to escape from autophagosomes [145, 146]. The importance of autophagy in limiting *L. monocytogenes* replication has been demonstrated *in vivo*, as mice deficient in autophagy exhibit increased bacterial load and decreased survival following infection [147]. The above-mentioned family of GTP-binding proteins again features prominently in autophagy as well*:* Gbp1 directs ubiquitin-associated *L. monocytogenes* to the autophagy machinery via binding to autophagy receptors [148–150]. To our knowledge, autophagy itself has never been examined as a function of age, not in humans or in mice; thus nothing is known about the role of autophagy in human neonatal listeriosis.

### 3.3. Fate of the* Listeria*-Infected Monocyte

*L. monocytogenes* induces cell death in multiple immune and nonimmune cell types (reviewed in [89]). Of all the cell death pathways induced by *L. monocytogenes*, T lymphocyte apoptosis is the best understood. *In vivo*, *L. monocytogenes* infection of mice is followed by rapid, synchronous, and extensive depletion of lymphocytes surrounding the periarteriolar lymphoid sheaths (PALS) in the spleen [27, 151]. The death of T lymphocytes in the PALS induced by *L. monocytogenes* is apoptotic in nature and precedes activation of T cells [152]. Importantly, the dying lymphocytes are not themselves infected with *L. monocytogenes*, indicating that apoptosis is caused by a factor extrinsic to the dying cell [88, 153, 154]. Dendritic cells can also respond with apoptosis to infection with *Listeria* (reviewed in [89, 155]). Most of the known pathways for the induction of apoptosis (Fas/FasL signaling, TNF-RI signaling, and perforin) were however shown not to be relevant in the development of the apoptotic lesions following infection of mice with *L. monocytogenes*. Only TNF-related apoptosis-inducing ligand (TRAIL) deficiency/ soluble DR5 (TRAIL antagonist), type I interferon receptor deficiency (IFNABR−/−), and granzyme deficiency [37, 38, 156–158] reduced T cell apoptosis *in vivo* following infection, suggesting they are involved. Treatment with type I interferon primes resting lymphocytes to undergo apoptosis induced by LLO [37]. Murine DCs and macrophages infected with *L. monocytogenes* produce massive amounts of type I interferon [43, 94, 159]. And IFN-abR −/− mice are more resistant to *L. monocytogenes* infection and display reduced apoptosis of splenic lymphocytes [37, 38]. The direct positive correlation between the strength of type I interferon induction, apoptosis, and virulence of particular strains of *L. monocytogenes* in mice further supports the importance of type I IFN for *Listeria*-induced apoptosis [160]. The proapoptotic effect of type I interferon on lymphocytes negatively influences the murine host systemic immune response to *L. monocytogenes* following infection, likely via induction of IL-10 [37, 161].

Data regarding the mechanisms by which *L. monocytogenes* induces cell death of monocytes and macrophages are inconsistent and somewhat contradictory, with evidence for apoptosis as well as pyroptosis, and necrosis [89]. Importantly, when *L. monocytogenes* kills the infected monocytes by necrosis, it is rendered less virulent [114]. Caspase-1-dependent cell death (pyroptosis) also reduces bacterial survival [115, 162, 163]. Thus, to promote its pathogenesis, *L. monocytogenes* must avoid killing infected monocytes via either necrosis or pyroptosis [109] and instead promote apoptosis [89]. Neonatal monocytes respond to innate stimulation with apoptosis at higher frequency [164], but this difference was detected following LPS stimulation. Nothing at all is known about the type of cell death induced in human neonatal monocytes infected with (or exposed to) *L. monocytogenes.*

### 3.4. Regulation of Cell-Autonomous Immunity in the Monocyte

Recent evidence suggests that epigenetics may play a role in regulating cell autonomous immunity. The transcriptional status of a gene is tightly linked to the structure of chromatin; transcriptional regulation of gene expression can be achieved via epigenetic regulatory mechanisms [138]. *L. monocytogenes* is known to reprogram host chromatin structure during infection to benefit its own survival (reviewed in [135, 138]). For example, *L. monocytogenes* induces acetylation of histone H4 as well as phosphorylation and acetylation of histone H3 specifically at the IL-8 promoter, leading to its downregulation in a p38 MAPK- and MEK1-dependent manner [165]. However, modulation of the monocyte epigenome can also work to the benefit of the host following for example BCG vaccination [166]. Neonatal mice are in fact completely protected from an otherwise lethal dose of *L. monocytogenes* if given BCG prior to infection with *L. monocytogenes* [57, 58]. As neonatal immunization of human newborns with BCG reduces neonatal mortality unrelated to tuberculosis, that is, nonspecifically [167], it may well be that regulation of cell autonomous immunity to *L. monocytogenes* is mediated via changes in epigenetics. While it is known that epigenetic modifications of immune-related genes vary with age [168], the role of epigenetics in cell autonomous immunity to *L. monocytogenes* remains hidden for now.

## 4. Conclusion and Outlook

Age-dependent differences in systemic innate and adaptive immunity to infection with *L. monocytogenes* very likely play a key role in the increased morbidity and mortality of the newborn. Several possibly relevant innate and adaptive immune response differences between newborn and adult have already been delineated; however few of these have been assigned clear functional roles in the host defence against *L. monocytogenes* (Table 1). Cell autonomous immunity seems particularly relevant following infection with *L. monocytogenes*; as the main target, the monocyte, is also centrally important to innate as well as adaptive systemic immunity to listeriosis. Thus, the outcome of infection of the monocyte is likely of paramount significance to systemic immunity of the host. However, currently nothing at all is known about age-dependent differences in cell autonomous immunity of the monocyte to infection with *L. monocytogenes* (Table 2). Given the many differences between murine and human listeriosis, studies aimed at identifying the molecular mechanisms relevant to age-dependent differences in cell autonomous immunity to infection with *L. monocytogenes* cannot indiscriminately be extrapolated from mouse to humans but will need to be conducted or at least confirmed in primary human monocytes. Identifying these aspects is likely to produce insights into not only pathogenesis but also interventions. Furthermore, the same age-defined high-risk period of severe listeriosis in the human (0–6 weeks) also represents high-risk periods for other relevant pathogens such as herpes simplex virus and group B streptococcus [169–176]. Thus, delineating the underlying mechanisms responsible for age-dependent risk for severe listeriosis potentially has broader implications.

## Figures and Tables

**Table 1:** Age-dependent differences in systemic immunity to *L. monocytogenes*.

Effector	Role in listeriosis	Neonatal mouse	Neonatal human
Neutrophils	Chemotaxis	?	Decreased
	Extracellular bacteria killing	?	?

Resident tissue macrophages	Production of chemokines	?	?
	Production of TNF*α*, IL-12p70, IL-18	Reduced IL-12p70	Reduced IL-12p70

Monocytes	Chemotaxis to infection site	Reduced	Reduced
	Differentiation to TipDCs and macrophages	?	?

Dendritic cells (DCs)	Antigen presentation	Reduced	?
	Production of IL-12p70	Reduced	Reduced

−CD8*α*+ DCs	Bacterial transport to PALS	?	?

−TNF*α*+ iNOS + DC (TipDC)	Production of TNF*α*, iNOS	?	?

NK cells	Production of IFN*γ*	?	?

CD4+ T cells	CD8 + Priming	?	?
	Cytokine production	Reduced	Reduced

CD8+ T cells	Bystander production of IFN*γ*	?	?

**Table 2:** Age-dependent differences in cell autonomous immunity to *L. monocytogenes*.

Effector	Role in listeriosis	Neonatal mouse	Neonatal human
Recognition of *L. monocytogenes *	(i) TLR/Myd88	?	?
	(ii) Cytosolic surveillance	?	?
	(iii) Inflammasome	?	?
Intracellular fate of *L. monocytogenes *	(i) Phagocytosis	?	?
	(ii) Autophagy	?	?
	(iii) IFN-inducible GTPases	?	?
Fate of *L. monocytogenes*-infected monocyte	(i) Apoptosis	?	?
	(ii) Necrosis	?	?
	(iii) Pyroptosis	?	?
